# Approaches in Characterizing Genetic Structure and Mapping in a Rice Multiparental Population

**DOI:** 10.1534/g3.117.042101

**Published:** 2017-06-05

**Authors:** Chitra Raghavan, Ramil Mauleon, Vanica Lacorte, Monalisa Jubay, Hein Zaw, Justine Bonifacio, Rakesh Kumar Singh, B. Emma Huang, Hei Leung

**Affiliations:** * Plant Breeding, Genetics and Biotechnology Division, International Rice Research Institute, Manila 1301, Philippines; † Discovery Sciences, Janssen R&D, Menlo Park, California 94025

**Keywords:** multiparental populations, imputation, recombination, QTL mapping, MPP

## Abstract

Multi-parent Advanced Generation Intercross (MAGIC) populations are fast becoming mainstream tools for research and breeding, along with the technology and tools for analysis. This paper demonstrates the analysis of a rice MAGIC population from data filtering to imputation and processing of genetic data to characterizing genomic structure, and finally quantitative trait loci (QTL) mapping. In this study, 1316 S6:8 *indica* MAGIC (MI) lines and the eight founders were sequenced using Genotyping by Sequencing (GBS). As the GBS approach often includes missing data, the first step was to impute the missing SNPs. The observable number of recombinations in the population was then explored. Based on this case study, a general outline of procedures for a MAGIC analysis workflow is provided, as well as for QTL mapping of agronomic traits and biotic and abiotic stress, using the results from both association and interval mapping approaches. QTL for agronomic traits (yield, flowering time, and plant height), physical (grain length and grain width) and cooking properties (amylose content) of the rice grain, abiotic stress (submergence tolerance), and biotic stress (brown spot disease) were mapped. Through presenting this extensive analysis in the MI population in rice, we highlight important considerations when choosing analytical approaches. The methods and results reported in this paper will provide a guide to future genetic analysis methods applied to multi-parent populations.

Traditionally, breeders and geneticists use biparental populations for mapping and varietal development. However, biparental populations have limited allelic variation as they only segregate for QTL that differ between the two parents. An alternate approach is to create multi-parental populations derived from elite parents, in which each line represents a combination of alleles inherited from multiple parents. Ideally, a diverse set of lines is generated simultaneously that can be readily used by breeders and geneticists. To achieve this, the selected parents must be (a) diverse in their traits, (b) good combiners, and (c) have elite features, which will result in new and favorable allelic combinations. Further, these diverse multi-parent lines serve as a genetic population to map QTL and to understand genetic interactions.

MAGIC populations are now becoming more common in a number of crop species such as rice, bread wheat, durum wheat, barley, and chickpea. Huang *et al.* (2015) provided a comprehensive review of the development and use of MAGIC populations, including brief descriptions of important populations under development. Breeders and geneticists have actively used and benefited from MAGIC populations, and several groups within the rice community are now also adopting this approach to develop trait-specific multi-parent populations (Bandillo *et al.* 2013; Leung *et al.* 2015).

Supported by the Generation Challenge program, the International Rice Research Institute (IRRI) initiated the development of multi-parent rice populations in 2008 to be used for both breeding and genetic studies. The four initial populations developed were: (i) MI (eight founders of the *indica* subtype); (ii) MAGIC PLUS (same eight founders as the MI population with two extra rounds of intercrossing); (iii) *japonica* MAGIC (eight founders of the *japonica* subtype); and (iv) the Global MAGIC (16 founders: the eight of the MI founders and the eight of the *japonica* MAGIC founders). The populations were generated from elite materials representing a diverse set of favorable traits. The MI population is the most advanced and has been studied in detail. At an early stage of population development (S4 equivalent to F5), Bandillo *et al.* (2013) mapped several traits on a small subset of the population, confirming that the lines were a well-recombined representation of the eight founders. Further, the populations were of interest to breeders due to the combination of their traits and favorable agronomic features leading to the selection of lines as prebreeding material. IRRI has since conducted several multi-environmental trials with a small subset of the MAGIC populations and continues to do so.

The variations in the design of MAGIC populations in terms of the number of founders, the mixing combinations, and the recurrent crossing designs followed by selfing, will all have an impact on the structure of the final population. Theoretical and simulation studies in rice have identified the potential benefits and levels of mapping resolution in various designs, but these have not yet been measured in a real population (Yamamoto *et al.* 2014). For example, these simulations suggest that a minimum of six cycles of crossing is required to significantly improve the mapping resolution in a population derived from eight parents. As might be expected from theory, increases in the number of recurrent cycles will be accompanied by an increase in the number of nonrecombined genome segments, as well as an initial decrease in segment length. For a population like the MI, which has not undergone any recurrent crossing “postmixing” of the founder genomes, the expected number of nonrecombined segments in 800 lines derived from an eight-way cross is ∼150 per line if markers are evenly spaced at 0.1 cM (Yamamoto *et al.* 2014). For comparison, Huang *et al.* (2009) estimated ∼33 recombinations per recombinant inbred line (RIL) in 150 lines derived from a biparental cross.

The predictions for the MAGIC structure based on high-density genotypes in simulation studies can now be verified using GBS data in these populations. Such low-coverage genotyping methods (Elshire *et al.* 2011) are popular due to their low costs and high throughput. However, they suffer from issues with missing data and genotyping error, the impacts of which are not yet well-characterized (Beissinger *et al.* 2013). In particular, it is unclear whether a powerful statistical methodology can offset some of the issues by capitalizing on genomic structure and observed data. Methods for genetic analysis (Huang and George 2011) and imputation (Huang *et al.* 2014) based on haplotype probabilities, calculated from founder information and the pedigree, have been developed specifically for multi-parent populations. These methods depend on the high-accuracy estimation of which alleles are contributed from each parent (founder allele inheritance), which is affected by population size, founder genetic similarity, marker density, and genotypic data quality. However, how to balance trade-offs in marker density and data quality resulting from a fixed budget remains to be determined.

Issues with genotyping and imputation will, in turn, have subsequent effects on QTL mapping analysis, introducing uncertainty and potentially identifying false positives. Hence, careful processing of GBS data is critical to correct for potential quality issues while maintaining the high density of desirable markers to identify regions of high LOD, define haplotypes, and characterize favorable founder alleles. Association mapping for several traits in the MI population have been performed (Bandillo *et al.* 2013), from which several large-effect QTL were detected. However, interval mapping using founder haplotypes with dense data has not yet been investigated using the rice MAGIC populations. Both mapping approaches have been applied in other populations such as barley (Sannemann *et al.* 2015), tomato (Pascual *et al.* 2016), durum wheat (Milner *et al.* 2015), and wheat (Mackay *et al.* 2014) and, in general, the combination allows the detection of QTL that might have been unaccounted for using one method alone.

This article summarizes the efforts in examining the MI S6:8 population to support and explore how these complex populations can be analyzed. A rice multi-parent population was used to address common aspects of MAGIC populations in crops, *e.g.*, low-coverage GBS, imputation, mapping traits, and identifying favorable alleles or founder haplotypes. The research assesses whether theoretical predictions of recombination are confirmed in an experimental population. It also discusses the criteria for filtering, paying careful attention to the impacts of genotyping error and missing data. Allowing a minimal level of missing calls in founders and lines, haplotypes were defined based on founder probabilities. The missing data were then imputed, and the resulting dataset was used to determine the number of observable recombinations and to map the QTL for eight traits of interest. QTL for agronomic traits (yield, flowering time, and plant height), physical (grain length and grain width) and cooking properties (amylose content) of the rice grain, abiotic stress (submergence tolerance), and biotic stress (brown spot disease) were mapped using interval mapping (R/mpMap) and association mapping approaches (R/GAPIT).

## Materials and Methods

### MAGIC population

The MAGIC population used for the analysis was derived from eight inbred elite founders of the *indica* subtype, which are widely adopted, high yielding, and tolerant to abiotic and biotic stresses. Detailed descriptions of the population can be found in Bandillo *et al.* (2013). In brief, the eight founders were intermated to derive 35 funnels with balanced contributions from each founder. The MI population was then derived by advancing 60 lines from each of the 35 eight-way F_1_s, resulting in a population consisting of 2100 lines. Approximately 2000 of these advanced intercross lines (AILs) are maintained, fixed at the S6 generation.

### Data curation

When dealing with large experimental trials, collecting phenotype data can be challenging in terms of replications and plot size, especially in multi-environment trials for complex traits. At the same time, seed increase and purity should be maintained to allow researchers to trace the material of interest. Data curation is important as it enables breeders to extract AILs with the desired combination of traits. Currently, all data generated at IRRI on the MAGIC populations including pedigree information are stored in the IRRI database B4R (Breeding for Rice). We have also provided a large amount of data in the supplement, with a guide to the supplement found in Supplemental Material, File S1. The data pipelines move from generating barcode, field layout, and field data collection on tablets. The data collected include information on field operations. Raw genotype data is stored for the validation of QTL and for future applications of improved analytical methods.

### Phenotyping

The population was phenotyped for various traits such as yield, flowering time, plant height, physical (grain length and grain width) and cooking properties (amylose content) of the grain, submergence tolerance, and brown spot disease. The above eight cases of QTL mapping results were selected to illustrate the mapping resolution. The trait analysis was conducted using PBTools (http://bbi.irri.org/products) and a broad-sense heritability, which is the proportion of the variation among entry means that is due to the variation in genotypic effects (File S2) for the traits, is reported.

#### Yield:

Yield trials were conducted for two dry seasons (2014 DS and 2015 DS) at IRRI under fully irrigated conditions using an augmented randomized complete block design. In the trials, the eight founders and four common checks [PSCBRC10 (IRRI 104), PSBRC 18 (IRRI 105), NSICRC222 (IRRI 154), and NSIC Rc132H (Mestizo 6)] were replicated three times. The number of hills harvested from each of the plots was recorded. Yield per plot (plot size: 2014 DS 96 m^2^ and 2015 DS 2.16 m^2^) was converted to tons/ha using the formula below:

Yield in tonnes/ha=(Actual Plot Yield/Effective Plot Size*)× 10 × Moisture Factor**

*Effective Plot Size(EPS)=Number of hills×distance between hills(m)×distance between rows(m)

**Moisture Factor(MF)=(100−%Moisture Content)/86

A one-stage linear model for augmented RCBD was used for analysis in PBTools (http://bbi.irri.org/products), treating genotypes as random with no relationships among lines. The BLUPs derived from the analysis were used for mapping. Details of the models used in the analysis are provided in File S2.

#### Flowering time and plant height:

Flowering time and plant height were measured in the 2015 DS yield plots. The field trial was conducted in the 2015 DS at IRRI in fully irrigated conditions using an augmented randomized design. The MAGIC lines, eight founders, and four checks were grown across three blocks, with checks and parents repeated in each block. Each line was transplanted in two rows of 27 hills and the number of days from sowing to 50% flowering was recorded. Plant height was measured at maturity in the same trial as the flowering time. A single environment analysis was conducted using PBTools, treating genotypes as a random factor to obtain BLUPs for both flowering time and plant height. The linear model for augmented RCBD was used for plant height and flowering time, with additional details given in File S2.

#### Grain physical and cooking properties (grain length, grain width, and amylose content):

Grain length, grain width, and amylose content (percentage of amylose by weight) were chosen to represent physical and chemical grain quality features. The grain length and width were measured using the FOSS cervitec, and amylose content estimation was based on the American Association of 643 Cereal Chemists Method 61–03 (AACC 1999). These grain traits were measured at IRRI’s Grain Quality and Nutrition Center. The grains of 1316 AILs and eight founders, harvested from lines grown in the field for seed increase in the 2012 wet season (WS), were used. Only a single sample from each plot was collected for quality assessment due to the high cost involved. The average grain length, average grain width, and percentage amylose content were used for mapping.

#### Submergence tolerance:

Submergence tolerance of the MAGIC lines was tested in the IRRI submergence ponds designed to hold water up to a depth of 1.5 m. The trial was conducted in a completely randomized block design composed of two replications. Seeds were directly sown at a high density (5 g/line) across six seedbeds per replicate. Checks with the *Sub1* QTL on chromosome 9 (Swarna-Sub1 and IR64-Sub1) and without (IR42, Swarna, and IR64) were used and represented in each of the seedbeds. The trial was subjected to complete submergence for 16 d, after which the water was drained. The survival of all lines was scored 7 d after drainage using a five-class visual score (IRRI 1996).

The nonparametric Friedman test, which is similar to the parametric repeated measures ANOVA, was used to derive the mean ranks using the Statistical Tool for Agricultural Research (STAR) software (http://bbi.irri.org/products).

#### Brown spot (Philippine isolate sm2):

Brown spot disease in rice is caused by the fungal pathogen *Bipolaris oryzae*. The fungus affects the plant at both seedling and adult stages, causing yield losses and negative effects to grain quality (Sato *et al.* 2015). The experiment was conducted in randomized complete block design with 36 blocks. All entries and founders were replicated three times and five checks were present in each block. Treatments and block were used as factors to derive BLUPs for QTL mapping. We tested the disease reaction of MAGIC lines, eight founders, and five checks (repeated in each block), to strain sm2, a local Philippine isolate of *B. oryzae*, at seedling stage. Fungal cultures were grown on potato dextrose agar plates and incubated at 25° for 10 d under alternating 12 hr in UV light and 12 hr in darkness to induce sporulation. The spores were scraped off and a spore suspension of 5 × 10^4^ spores/ml was used to infect the seedlings. The inoculated seedlings were placed in a humid chamber overnight and maintained for 7 d in a cool room at 22°. In susceptible plants, this produced brown lesions on young leaves. The phenotyping was conducted in the greenhouse at IRRI. The most infected leaf was evaluated 7 d postinoculation. Images of diseased leaves were taken under a fluorescent lamp light box using a regular digital camera. The camera was at set distance from the object, in this case the diseased leaf or lightbox. Images of infected leaves were bulk analyzed using a customized protocol run on ImageJ software to measure the diseased leaf area. The total leaf area and the area covered by the lesion were measured using a color threshold based on which percent of diseased leaf area was estimated. A single environment analysis was conducted using PBTools, generating BLUPs. Details of the model used are provided in File S2.

### Genotyping

A total of 1316 S6:8 lines and nine replicates of the founder lines were genotyped using GBS (Elshire *et al.* 2011). Full details of the GBS pipeline approach can be found in Glaubitz *et al.* (2014). Briefly, it consists of wet lab processing of samples accompanied by an informatics pipeline prior to making SNP calls. The 96-plex ApeKI GBS protocol was used, wherein sets of 96 samples per lane were sequenced on an Illumina HiSeq. The GBS pipeline was run by the Philippine Genome Center of the University of the Philippines using TASSEL software version 3.0.169 (Glaubitz *et al.* 2014). The sequence reads were aligned to the reference genome Nipponbare sequence MSUv7 to derive the physical positions of markers. The raw GBS data files are extremely large and require specialized bioinformatics pipelines. Indeed, important considerations in running the GBS data pipeline include the transfer and storage of such data. An Amazon Elastic Cloud (Amazon EC2) instance was used in this study to enable uninterrupted analyses.

### Marker filtering post-GBS pipeline

**Figure 1:**
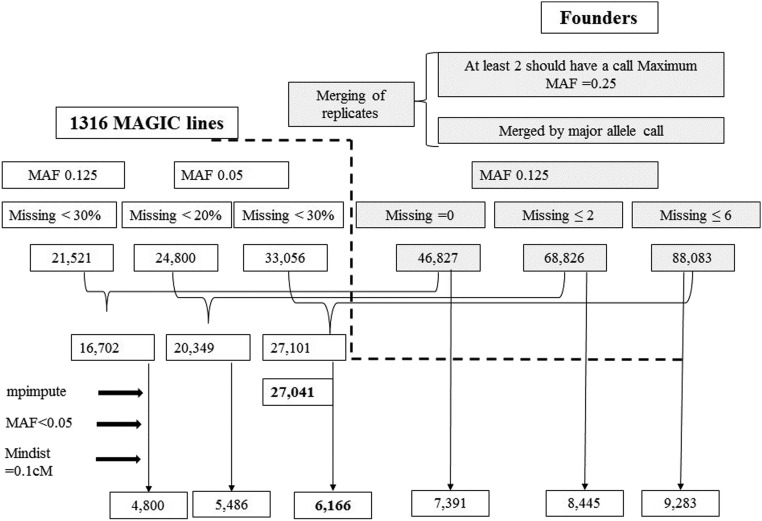
Filtering for SNP markers from GBS marker data in founders and lines (post-GBS pipeline). The scheme shows the use of different criteria resulting in multiple sets of SNP marker sets, emphasizing the impact of filtering criteria on downstream results. GBS, genotyping by sequencing; MAF, minor allele frequency; Mindist, minimum distance between any two markers; SNP, single nucleotide polymorphism.

Postprocessing steps were applied to the genotype data, imposing various criteria, to extract multiple SNP sets in order to compare the effect of different numbers and quality of SNPs on detecting recombinations (Figure 1). The SNP markers in founders and lines were filtered separately and the filtered SNPs common to both lines and founders were used for analysis. To filter the founders, replicates were first merged by ensuring that at least two had observed calls, with up to a quarter of the replicates allowed to have an alternate call. The alternate call within replicates of founders reflects genotyping errors. The most common allele was then taken as the founder call. Next, SNP markers with a minor allele frequency (MAF) of ≥ 0.125 (*i.e.*, one of the eight founders) at three levels of missing call rates across the eight founders (no missing calls, allowing up to two founders to have missing calls, and allowing up to six founders to have missing calls) were extracted. To filter the lines, a similar criterion was applied at two levels of MAF (0.125 and 0.05). The SNPs obtained at MAF = 0.125 were filtered, allowing only those with < 30% missing data, while the SNPs obtained at MAF = 0.05 were further filtered allowing two levels of missing data (< 20 and < 30%). Finally, the filtered sets of SNP markers of founders and lines were merged in three combinations to obtain the markers present in both lines and founders. Marker data from the lines for the three sets of markers filtered in the founders only were also extracted. In total, six sets of filtered SNP data were examined.

### Imputation

Genotypes were imputed using the “mpimpute” function in R/mpMap (Huang *et al.* 2014). This method first estimates the probability that observed lines inherit alleles from each founder based on a pedigree-informed hidden Markov model (HMM), then audits the observed line genotypes for each founder to impute founder genotypes. Once the founders have been imputed, individual genotypes are imputed based on the updated HMM probabilities. Missing calls in the lines and founders were first imputed using the R/mpimpute function, and then the markers were filtered based on MAF. The imputed data were used for estimating recombination levels and interval mapping of multiple traits in the set of 1316 genotyped AILs.

### Defining haplotypes and estimating founder probabilities

This study was interested in estimating the probability that a given region derives from each founder, as they can be used for defining haplotypes and for QTL mapping. In estimating these probabilities, several sets of SNP markers were used, with the attempt to saturate the genome with markers spaced no closer than 0.1 cM. Larger sets of SNPs are based on less stringent filtering criteria, hence, are of lower quality. In this study, six sets of SNP markers, which were derived using different criteria (Figure 1), were tested.

For interval mapping, the map distance had to be represented in centimorgans, hence we used a linkage map derived from the physical map based on the conversion factor of 1 cM = 250 kb. The estimation of the equivalent centimorgan to bases was made using information from published high-quality, high-density genetic IRMI (accession ID irmi-2003, http://archive.gramene.org/db/markers/marker_view?action=view_map_set&map_set_acc=irmi-2003) and physical IRGSP map (available in Gramene as accession ID wig2005b; http://archive.gramene.org/db/cmap/map_set_info?map_set_acc=wig2005b) of SSRs in the Nipponbare rice genome. SSRs in common between the genetic and physical maps (1224 in all) were determined, the respective coordinates (in centimorgan and base pair positions) tabulated, and the distances between SSRs (in centimorgan and base pairs) were computed for the entire genome. The average base distance–centimorgan ratio was computed between adjacent markers, per chromosome, and for the entire genome (File S3), resulting in the genome-wide ratio (∼250 kb/cM) as the kilobase–centimorgan estimator.

Founder probabilities were estimated at all markers using the “mpprob” function in R/mpMap (Huang and George 2011) with program = qtl. The number of recombinations was then calculated at all marker positions (spaced > 0.1 cM apart) using a forward–backward dynamic programming algorithm with a penalty of five for switching between founders (File S4). The penalty acts as a disincentive to call recombination breakpoints; at its lowest values, we take the most probable founder at each genomic location, while as the penalty becomes large we expect the number of recombinations to decrease.

### Mapping

#### Genome-wide association mapping (GWAS):

GWAS mapping was carried out using R/GAPIT (Lipka *et al.* 2012). The compressed mixed linear model (MLM) method was applied for detecting QTL associated with the trait (Zhang *et al.* 2010). A filtered set of 27,041 markers (Figure 1) across the 1316 MI lines was used for analysis. A kinship matrix based on the marker data was generated within the analysis. The default criteria implemented in GAPIT was used, with a significance threshold of 0.0001.

#### Interval mapping:

Interval mapping was conducted using the function “mpIM” from R/mpMap. Simple interval mapping (SIM) was performed using BLUPs as response for the majority of the traits. The significance threshold for SIM was set to p-value < 0.0001. Next, the function “fit” was used to simultaneously estimate the effects of all QTL by fitting all the detected QTL in a single model. Note that this means that some QTL are no longer significant once all are included simultaneously. In this study, two separate final models, one based on percent variance and one based on p-value, were considered. The function “qindex” was used to select QTL based on two criteria: (a) percent variance > 2 and p-value < 0.05, and (b) only p-value < 0.0001 to include for fitting the final model. LD was estimated between flanking markers for QTL using the function “mpcalcld” in R/mpMap (Huang and George 2011). This uses a multi-allelic measure for LD as described in Huang *et al.* (2012).

### Data availability

The authors state that all raw data pertaining to the experiments has been submitted to the journal. A guide to supplemental files and raw data submitted to the journal can be found in File S1. Raw genotype data can be found in http://snpseek.irri.org/_download.zul. IRB is not applicable in our case. Downloadable files are available at URLs: Hapmap data (https://s3-ap-southeast-1.amazonaws.com/oryzasnp-atcg-irri-org/pub-data/MAGIC-Raw-genotype-data-Raghavan-2017.zip) and VCF data (https://s3-ap-southeast-1.amazonaws.com/oryzasnp-atcg-irri-org/pub-data/MAGIC-vcf-all-chromosomes.zip).

## Results and Discussion

### GBS data filtering

The postprocessing of GBS data was considered in detail given its tendency for genotyping errors. The SNP data of each founder replicate was compared against its 14 × sequence data in the 3 K panel (Alexandrov *et al.* 2014). On average, the GBS calls and 14 × calls differed by 1.7%. This information was used to assume less than a 2% error in genotyping. The GBS raw data, those which were run through the standard GBS pipeline TASSEL 3, were used to generate SNP calls that served as the primary GBS dataset (as compared to the filtered datasets from postprocessing). The filtering of SNPs significantly reduced the original number of SNPs. Founder filtering resulted in three sets of SNPs (46,827, 68,826, and 88,083), MAGIC line filtering resulted in a further reduction of SNP markers (21,521, 24,800, and 33,056), and finally the merging of founders and lines resulted in three sets of common markers (16,702, 20,349, and 27,101), as shown in Figure 1. SNP data were also extracted from lines corresponding to markers filtered in the founders only (46,827, 68,826, and 88,083) (see dotted line in Figure 1). The six datasets derived consisted of 16,702, 20,349, 27,101, 46,827, 68,826, and 88,083 markers. These filtering criteria will allow researchers to select an appropriate set of SNPs, keeping in mind that the stringency of criteria will provide a more accurate but smaller set of SNPs. Hence, the choice of thresholds will depend on the goals for analysis, as interval mapping may require fewer markers than fine-scale association mapping.

### Imputation and marker spacing

To regain some of the information lost through filtering, the missing data for the founders and AILs were imputed. The function “mpimpute” (Huang *et al.* 2014) has been shown to have nearly 100% accuracy in founder imputation and > 90% imputation accuracy for a range of sample sizes, levels of missing data, and marker densities. At postimputation, SNPs with MAF < 5% in the AILs and with a minimum intermarker distance of 0.1 cM were filtered out.

As the filtering stringency decreased, the number of marker data points increased. In the first three data sets (16,702, 20,349, and 27,101), the marker data of lines were filtered for missing data and MAF at the first stage prior to imputation, while no filtering was applied to the line marker data in the following three datasets (46,827, 68,826, and 88,083) (Figure 1).

In order to select a dataset for mapping, an intermediate filtering criterion was used. The largest set of markers was selected for lines where filtering was applied prior to imputing (33,056) and was combined with the largest dataset for founders (88,083), resulting in 27,101 markers common to lines and founders. It was noted that missing data were high because the replicated founder data (missing ≤ 6) were used for the first stage of filtering. Imputation of missing line and founder marker data followed by filtering for MAF < 0.05 resulted in complete data (no missing data points) for 27,041 markers, which were used for GWAS. To extract markers for interval mapping, the 27,041 markers were binned such that no two markers were closer than 0.1 cM or 25,000 bp, which resulted in 6166 markers. All interval mapping performed in this study was conducted on this set of 6166 markers. On average, the intermarker distance was 0.24 cM; 66% of the markers had an intermarker distance of < 0.2 cM and 98% of them were within 0.65 cM.

### Founder probabilities and levels of recombination

**Figure 2:**
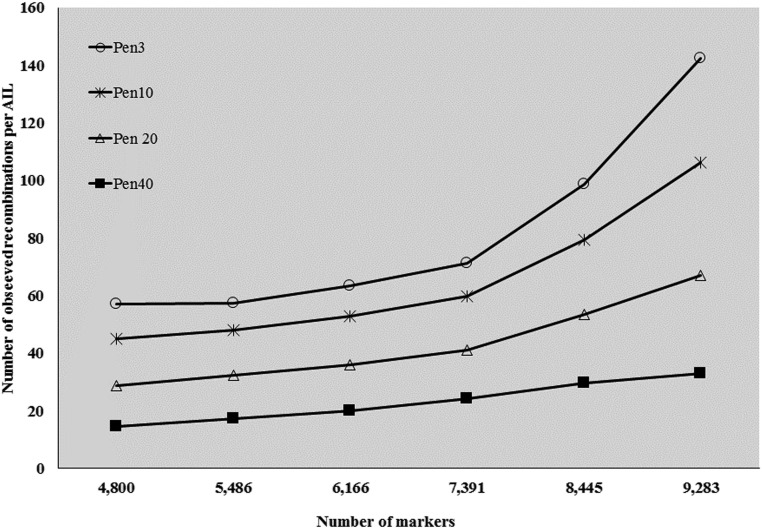
Graph showing the trend in average number of observed recombinations per line of *indica* MAGIC (MI) population. The estimates are made at varying numbers of markers (*x*-axis) and penalty (Pen) levels. The *x*-axis indicates the number of single nucleotide polymorphism (SNP) markers used for estimating recombinations/marker sets (see Figure 1). Recombinations were estimated at a genotyping error probability of 0.3. AIL, advanced intercross lines.

Founder probabilities and levels of recombination were estimated using the imputed marker sets that were filtered for MAF (4800, 5486, 6166, 7391, 8445, and 9283) (Figure 1). As the number of markers increases, the chance of observing genotyping errors and, hence, overestimating the number of recombinations estimated also increases. This trend is apparent at varying marker levels across the filtered data sets. To counteract the trend, this study introduced a penalty for shifting between founders and considered the effect of varying levels of penalty on recombination estimates (Figure 2). By filtering on the minimum distance between markers (mindist = 0.1 cM), the number of markers and consequently the number of estimated recombinations were reduced. It is important to note that the marker data are not uniformly dense since the maximum distance was not limited.

Between 63 and 71 recombinations were observed per AIL at the lowest penalty (3) and a probability of genotyping error of 0.03 using 6166 and 7391 markers, respectively (Figure 2). Also, the number of recombinations increased with the number of markers, and increasing the penalty resulted in fewer estimated recombinations. At all the penalty levels tested, the number of recombinations did not change much as the number of markers increased from 4800 to 6166, but a steeper increase in the following three marker sets (7391, 8445, and 9283) was observed. In this study, as the penalty increased, the slope decreased with increasing marker numbers. Ideally, one might expect the number of recombinations to stabilize as the genome is saturated with markers. As the least stringent criteria produced a set of 9283 markers, there is still scope for greater saturation. In comparison to these results, Huang *et al.* (2009) reported 33.8 recombination breakpoints per RIL derived from a cross between *Oryza sativa* 93-11 (*indica*) and Nipponbare (*japonica*).

This study found slightly more recombinations than the simulations reported by Yamamoto *et al.* (2014), though there are a number of possible reasons for this difference. To saturate the genome (373,245,519 bp) (http://rice.plantbiology.msu.edu/annotation_pseudo_current.shtml) with markers every 0.1 cM, ∼15,000 markers would be needed (assuming 0.1 cM equals ∼25,000 bp). With the current dataset, we were able to extract a maximum of 9283 markers. Simulation experiments (population size *n* = 800; genome size 1526.8 cM; bin 0.1 cM; cycles 0) reported ∼150 genome segments per individual (Yamamoto *et al.* 2014).

Due to the increase in the number of recombinations observed beyond 7391 markers, we decided to select 6166 markers for mapping. This choice of core set was also based on the intermediate filtering criteria, and the fact that no SNP data were missing in the founders for these markers. While it would be interesting to see the effect of filtering and imputation on mapping, it would be best to investigate this in simulated data.

Some of the issues we have seen with genotype errors, and increases in recombination breakpoints with additional markers, may be due to our use of a linkage map based directly on the physical map. Estimation of recombination (centimorgan distance) from physical genome size is best done in a proper mapping population, as the values are dependent on the genomes of the parents being used in the cross. However, comparative *O. sativa* genome sequencing publications (Yu *et al.* 2005; Schatz *et al.* 2014; Sakai *et al.* 2014), show that various rice cultivars have very high genome similarity (and chromosome colinearity) across major subpopulations (> 95%), which gives us confidence in using a single estimation of recombination for *O. sativa*. The availability of a high-quality genome sequence in rice, and high-density genetic maps using sequence-based markers (SSR), have enabled a reasonable estimation of the physical-to-centimorgan distance conversion in rice. This process will be least appropriate in regions where the MI founders differ from the reference, and due caution should be exercised for any QTL that appear in such regions.

In general, it must be kept in mind that the choice of filtering thresholds will influence downstream analysis. It is clear that larger populations and dense markers are required to detect small-effect QTL or recombinations, but this also entails a higher cost in genotyping. Lower-cost genotyping technologies, while producing larger numbers of markers, may be prone to more errors and, hence, will require more stringent filtering, which in turn reduces the number of markers. Statistical methods such as applying penalties and marker imputation as discussed above may correct this to some extent, but these must be used with caution.

### Phenotype analysis and QTL mapping

The summary statistics of the traits in the population and founders are shown in Table S1. Generally, in cases when there were contrasting alleles within the founders for traits, transgressive segregation was observed. For most traits, we observe a continuous range of values among founders, except in case submergence tolerance where only Sambha Mahsuri-Sub1 is tolerant.

The imputed and filtered marker data were used for QTL mapping, with a linkage map generated from the physical map of the Nipponbare reference genome. The sequence length in the pseudomolecule release 7 (MSUv7) is 373,245,519 bp. This roughly converts to 1492 cM, which is comparable to map lengths reported earlier (Harushima *et al.* 1998). Similar procedures for interval mapping were followed for all eight traits considered, with BLUPs taken as response in the genetic model. We note that this may not be ideal in the case of low heritability traits, but our traits all have medium to high heritability. We report peak widths for both interval and association mapping, but note that these are not exactly comparable, since for association mapping we take the peak to include all markers significantly associated with the trait, whereas for interval mapping this is based on a 1 LOD drop-off. The average 1 LOD support interval of the QTL reported in this study is 2.83 cM (0.707 Mbp), the interval between flanking markers is 0.29 cM (0.072 Mbp), and the LD (*r*^2^) between flanking markers is 0.85 (Table S3).

#### Yield:

The BLUPs of yield for the founders ranged between 5.4 and 6.4 tons per hectare with Fedearroz 50, IR77186-122-2-2-3 (PSBRc 158), and IR64633-87-2-2-3-3 (PSBRc82) being the top three yielders. The BLUPs of yield for the MAGIC lines ranged from 4.4 to 8.6 tons per hectare, with nearly 20% (286 of the 1316) of the MI entries having greater yield than the founders. The high-yielding checks NSCIRc 222 (IRRI 154) and NSIC Rc132H (Mestizo 6, hybrid variety) yielded 6.7 and 7.9 tons per hectare, respectively, in the conducted trials. The heritability for this study was 54%. SIM detected four QTL on chromosomes 2, 3, 7, and 8. The phenotypic variance explained by the final model with percent variance > 2% and p-value < 0.05 was 10.13%, supported by a QTL on chromosome 3 (4.19 cM; 1.27 Mbp) and another QTL on chromosome 8 (104.62 cM; 26.2 Mbp) (Figure S1). The phenotypic variance of the QTL on chromosome 3 was 7.68% and that on chromosome 8 was 3.17%. The phenotypic variance explained by the final model with p-values < 0.0001 was 10.13%, and was also supported by the two QTL on chromosomes 3 (4.19 cM; 1.27 Mbp) and 8 (104.62 cM; 26.2 Mbp). The results of SIM for 2014 DS or 2015 DS also detected these QTL on chromosomes 3 and 8.

Association mapping detected significant associations on chromosomes 3 and 8. The SNP markers on chromosome 3 at positions 1,270,943 and 1,343,246 bp were the top two hits with p-values 1.74E−14 and 3.09E−14, respectively, and with *R*^2^ equal to 4.9%. On chromosome 8, one marker on position 26,573,952 bp was detected to be significant at p-value 1.14E−05 and with an *R*^2^ of 4.9%.

The QTL on chromosome 3 (4.19 cM; 1.27 Mbp) was also detected as being significant for flowering time (Table S2). The QTL on chromosome 8 colocalized with the QTL that was reported to be linked to grain weight per plant and source activity (source QTARO: http://qtaro.abr.affrc.go.jp) (Yamamoto *et al.* 2012). In this study, small-effect QTL for yield were detected, which will be validated. SIM also detected significant QTL on chromosomes 2 and 7 (Table S2) that colocalized with the QTL for plant height qPH-2 (QTARO) and the QTL for 1000-grain weight *gw7* (Li *et al.* 2000), respectively. Since yield is a complex trait, it may be a better approach to study the marker–marker and marker–trait interactions rather than to validate each small-effect QTL independently.

#### Flowering time:

The flowering time of the eight founders ranged between 85 and 101 d, with IR77298-14-1-2-10 flowering the earliest and Sambha Mahsuri-Sub1 flowering the latest. Flowering in the MAGIC lines ranged from 84 to 106 d. The heritability for flowering time was 49%. Eight significant QTL were detected using the SIM approach (Table S2). The final model with percent variance > 2 and p-value < 0.05 detected four QTL on chromosomes 1, 3, 6, and 7 (Figure S2), and explained 22.36% of the variance. The QTL on chromosome 3 (4.19 cM; 1.27 Mbp) accounted for 17.51% of the phenotypic variance. The final model with p-value < 0.0001 detected QTL on chromosomes 3, 5, and 6, explaining 22.97% of the variance.

Association mapping detected 73 markers associated with flowering time on chromosome 3. The SNP markers on chromosome 3 at positions 1,343,246 and 1,270,943 bp (which were also flanking markers detected by interval mapping) were the most significant markers with p-values of 3.57E−32 and 4.34E−32, respectively, and an *R*^2^ of 12.9%. The QTL detected on chromosome 3 (between 1,270,943 and 1,343,246 bp) colocalized with the *Hd9* (heading date) and *dth3* (days to heading/drought tolerance) QTL (Lin *et al.* 2002). Association mapping also detected three markers (p-value < 0.003) between ∼31.9 and 31.97 Mbp on chromosome 1, which are close to the QTL detected at 127.84 cM (31.97 Mbp). The marker at position 31,971,239 bp was the most significant and is also a flanking marker reported by SIM (Table S2).

The QTL on chromosome 1 detected by SIM (153.1 cM; 38.29 Mbp) colocalized with the QTL reported for panicle/flower morphology and development as well as for plant height (*sd1*) (McNally *et al.* 2009) (Table S2). In this study, SIM detected a second QTL on chromosome 1 (127.84 cM between 31.9 and 31.97 Mbp) (Table S2) which colocalized with a flower morphology QTL. The two QTL on chromosome 1 are separated by 25 cM (6.31 Mbp). The QTL on chromosomes 6 and 7 colocalized with the days-to-heading QTL *Hd3b* (Yano *et al.* 2001) and *Hd7*, respectively (QTARO). A comparison of QTL for yield and QTL for flowering time shows the QTL in proximity (Table S3), suggesting possible correlations between the traits.

#### Plant height:

In this trial, the plant height of the MAGIC founders ranged from 86 (IR64633-87-2-2-3-3 (PSBRc82) to 112 cm (IR 4630-22-2-5-1-3), while the height of the lines ranged from 73 to 136 cm. The heritability for plant height was 70%. SIM detected 15 QTL (Table S2). The final model with percent variance > 2% and p-value < 0.05 was explained by the QTL on chromosomes 1, 3, 4, 5, 6, 7, 11, and 12 (shown in bold in Table S2) accounting for 26.19% of the variance. The final model with p-value < 0.0001 explained 23% of the phenotypic variance, due to the QTL on chromosomes 1, 3, 5, and 11. The QTL on chromosome 1 (153.71 cM; 38.44 Mbp) (Figure S3) explained 9.8% of the variance and colocalized with *sd1* (McNally *et al.* 2009). It should be noted that a second QTL was detected on chromosome 1 by SIM at 38.16 cM (9.55 Mbp).

GWAS detected 61 markers on chromosome 1, including flanking markers from the interval mapping between 35 and 41 Mbp, to be significantly associated with an *R*^2^ of 17–18%. The second largest QTL, which explained 8.5% of the variance, was detected on chromosome 3 at 4.68 cM, ∼0.5 cM (0.12 Mbp) from the yield/flowering QTL. This QTL on chromosome 3 was also detected by GWAS with 17 markers significantly associated, although they were spread over 21 Mbp. There are possible interactions between the loci controlling yield, plant height, and flowering time. The QTL on chromosome 6 (56.83 cM; 14.35 Mbp) colocalized with a previously reported plant height QTL *qPH2-6-1* (Cui *et al.* 2004), which has also been indicated in an interaction between tiller number, plant height, and heading date.

#### Grain length:

The grain length of the founders ranged from 5.09 to 6.97 mm with IR77298-14-1-2-10, IR64633-87-2-2-3-3 (PSBRc82), and IR77186-122-2-2-3 (PSBRc 158) having long grains (6.6–7.00 mm). Grain length in the examined MAGIC population ranged from 5.1 to 7.3 mm. SIM detected 10 QTL. The final model with percent variance > 2 and p-value < 0.05 explained 49.05% of the variance, with the QTL on chromosomes 3 (65.68 cM, 16.64 Mbp) and 7 (98.73 cM; 24.70 Mbp) accounting for 31.51 and 13.66% of the phenotypic variance, respectively (Table S2). The QTL on chromosomes 3 and 7 were also fit by the final model with p-value < 0.0001 and explained 46.73% of the variance (Figure S4).

The QTL on chromosome 3 was also detected by association mapping. There were 57 significant markers between 16.2 and 21 Mbp. This was also previously reported in an earlier study on a subset of the MI early generation (S4) population (Bandillo *et al.* 2013). This QTL is known to colocalize with the QTL for grain length (*qGL-3*), grain width (gw3.1), and length by width ratio (*qLWR-3*) (Yu *et al.* 2011) (source QTARO). It is also located near the grain size QTL *GS3* (Fan *et al.* 2006). In the larger (1316 lines) S6:8 population, 53 markers at ∼24 Mbp on chromosome 7 were detected to be associated with grain length. This QTL on chromosome 7 has been previously reported to be linked to the grain width QTL *grb7-2* (source QTARO) or grain size (Shao *et al.* 2012).

#### Grain width:

The grain width of the founders ranged from slender (1.85 mm) to broad (2.5 mm). Sambha Mahsuri-Sub1 and IR77186-122-2-2-3 (PSBRc 158) both have slender grains compared to the broad grains of IR4630-22-2-5-1-3 and IR45427-2B-2-2B-1-1. In the MI population, grain width ranged from 1.7 to 2.8 mm. SIM detected 29 QTL. The final model with percent variance > 2 and p-value < 0.05 explained 57.18% of the variance based on 19 QTL (shown in bold in Table S2). Seven QTL on chromosomes 1, 2, 3, 5, 7, and 8 (Table S2) were detected by the final model with p-value < 0.0001 and accounted for 47.08% of the phenotypic variance.

Association mapping detected 39 markers (4.3–5.6 Mbp; *R*^2^ 46.5%) on chromosome 5 that are associated with grain width. The marker at 5,391,586 bp, which is also the right flanking marker (Table S2) on chromosome 5, was the most significant. The QTL on chromosome 5 (4.9–5.4 Mbp) colocalized with *qGW-5* (Weng *et al.* 2008). GWAS also detected 49 markers on chromosome 7 (24.4–24.8 Mbp) to be associated with grain width. The QTL on chromosome 7 (97.6 cM; 24.42 Mbp) colocalized with the grain width QTL *grb7-2* (source QTARO) or grain size (Shao *et al.* 2012). This QTL explained the most phenotypic variance (16.58%). It is situated 1.13 cM (0.28 Mbp) away from the QTL detected for grain length in this study (Table S2). The QTL on chromosome 8 (105.95 cM; 26.54 Mbp) (Table S2) was detected by both interval and association mapping (26.3–27.3 Mbp) approaches and colocalized with the grain width and yield potential QTL *GW8* (Wang *et al.* 2012). The QTL on chromosomes 5, 7, and 8 (Figure S5) have also been previously reported in the MI population (early generation S4) (Bandillo *et al.* 2013).

SIM detected a fourth significant QTL on chromosome 3 at 97.21 cM (interval 24.48 and 24.53 Mbp), which colocalized with the grain length or width-related QTL (*qGL-3*, *gw3.1*, and *qLWR-3*) and with the QTL linked to grain weight (*gw3a*) and yield (*Yd3-13*) (source QTARO). GWAS (16.7–16.8 Mbp) and SIM detected a QTL for grain width on chromosome 3 at 64.33 cM (16.3 Mbp) (Table S2) which is 1.35 cM (0.34 Mbp) away from the QTL for grain length (Table S2) detected in this study. The locations of the QTL on chromosome 3 for yield, flowering time, plant height, and grain length and width suggest possible links among these traits.

#### Amylose content:

Amylose content is a key cooking quality trait that determines consumer preference. The amylose content (percent by weight) in grains of the founders fell into a narrow intermediate type category between 19.8 and 24.6%. Amylose content in the rice grains of the MAGIC population ranged from 11 to 25%. SIM detected 12 QTL with the most significant QTL found on chromosome 6 (Table S2). The final model with percent variance > 2 and p-value < 0.05 included five QTL (Table S2), explaining 53.62% of the phenotypic variance. Only one large QTL on chromosome 6 was fitted by the final model with p-value < 0.0001, accounting for 49.92% of the phenotypic variance. The QTL on chromosome 6 which localized within the *waxy/qAC-6* locus (1.69–1.72 Mbp) alone accounted for 49.92% of the phenotypic variance (Figure S6).

Association mapping detected 123 significant markers on chromosome 6 between 0.36 and 2.8 Mbp, with a marker at 1,760,469 bp being the most significant. It is noted that this QTL (chromosome 6: 6.33 cM; 1.72 Mbp) was closely located to a QTL for flowering time (chromosome 6: 7.75 cM; 2.07 Mbp) (Table S2). A second QTL on chromosome 6 (68.99 cM; 17.39 Mbp) detected by SIM colocalized with an amylose content QTL *amy6-1*, which is located in proximity to a plant height QTL (56. 83 cM; 14.35 Mbp) detected in this study. The QTL for yield, flowering time, and plant height have also been reported at this locus (source QTARO). Although several other (besides the ones detected on chromosome 6) QTL were detected in this study, only those on chromosomes 1 (154.39 cM; 38.61 Mbp) and 5 (detected only by SIM at 91.51 cM; 22.91 Mbp) colocalized with previously detected QTL associated with eating quality.

#### Submergence tolerance:

Sambha Mahsuri-Sub1 was the only tolerant founder used. The tolerant checks Swarna-Sub1 and IR64-Sub1 were used alongside the susceptible checks Swarna, IR64, and IR42 in the submergence trials. Only ∼7% of the lines were tolerant to 16 d of flooding (*i.e.*, survived 7 d after draining). SIM detected seven QTL on chromosomes 1, 3, 5, 7, and 9. The final model with percent variance > 2 and p-value < 0.05 detected QTL on chromosomes 7 and 9 and explained 23.97% of the variance, with the largest QTL being the *Sub1* QTL (22.44%) (Table S2). The final model with p-value < 0.0001 detected one large QTL on chromosome 9 at 27.15 cM (7.08 Mbp). The major *Sub1* QTL (chromosome 9: 27.15 cM; 7.08 Mbp) was mapped (Figure S7) within a 1 LOD support interval 0.69 cM (0.17 Mbp) and 31.9 kb between markers at 7,045,612 and 7,077,542 bp. The QTL *SUB1A* is linked to culm/shoot growth trait (Source QTARO). A second QTL on chromosome 9 (81.94 cM; ∼20.7 Mbp), which colocalized with the QTL for flowering (Os*RRMh*) and drought tolerance/root length (*mrl9a*) (Source QTARO), was also detected by SIM in this study. The QTL detected by SIM on chromosome 1 (between 2,349,219 and 2,502,008 bp) colocalizes with *qSUB1.1* recently reported in a FR13A by IR 42 RIL (Gonzaga *et al.* 2016).

Association mapping detected 100 significantly associated markers, with the top 10 markers located between 6.1 and 6.19 Mbp on chromosome 9. The QTL on chromosome 7 (82.03 cM; 20.5–20.8 Mbp) (Table S2) colocalized with the days-to-heading QTL *dth7.1* (Gao *et al.* 2014) (Source QTARO). Toojinda *et al.* (2003) reported a QTL for submergence tolerance on chromosome 7 (19,256,914–19,257,039 bp). It was noted in this study that a small proportion of lines are submergence tolerant, which may be due to interactions between several QTL and the major *Sub1* QTL detected in this study. The submergence tolerance mechanism is associated with suppressed shoot growth and delayed flowering (Septiningsih *et al.* 2013). The MAGIC population would be ideal to understand the interaction between the detected QTL and to identify lines with different combinations of these QTL.

#### Brown spot:

The parents exhibited variation in resistance to sm2, an isolate of *B. oryzae*. IR77298-14-1-2-10 was the most susceptible while Sanhuangzhan-2, Sambha Mahsuri-Sub1, and IR4630-22-2-5-1-3 were resistant. The BLUPS in the AILs ranged from 7 to 30 (resistant to susceptible). The heritability for brown spot was 40%. SIM detected a large QTL on chromosome 12 (76.71 cM; 19.30 Mbp) (Figure S8) and two others on chromosomes 4 and 8. Only the QTL on chromosome 12 was fitted by both the final model with percent variance > 2 and p-value < 0.05 and also the model with p-value < 0.0001. The phenotypic variance accounted for by both the final models was 34.42%.

GWAS mapping also detected the QTL on chromosomes 12 (19.3 Mbp) as highly significant (p-value = 3.62E−25). This QTL has been validated independently in other studies (biparental and 2 k diversity panel) at IRRI. The QTL interval (chromosome 12: 19,301,158–19,339,344 bp) colocalized with *qDLA-12-3* (QTARO), a QTL for leaf blast resistance. A second QTL on chromosome 12 was detected by SIM at 39.83 cM (10.08 Mbp), which colocalized with *qDLA-12-3* and is near the meta QTL yield under drought stress (MQTL) dty_12.1_ (Swamy *et al.* 2011). The QTL on chromosome 8 also detected by SIM colocalized with a drought tolerance QTL and a flowering QTL *qDTH8* (QTARO). Since brown spot disease is often observed in regions where there is water/soil stress, the QTL on chromosome 8 might be interesting to further investigate.

### Conclusions

This paper reports on analytical methods applied to understand the genetics of the MI population in rice. It shows how GBS data can be used to generate large numbers of SNP markers as well as the importance of: (a) filtering criteria, (b) imputation of missing data, and (c) accounting for genotyping errors in downstream analysis. We have shown how filtering criteria affect estimated recombination numbers. Datasets derived from low filtering stringencies have high recombination numbers based on theoretical assumptions. These high levels could possibly be due to genotyping errors, which we accounted for in the analysis. Replicating founders for sequencing is useful to ensure high confidence of SNP calls. An alternative is to have deep sequence data for the founders, which may be used to impute SNP marker calls in lines to improve coverage. When dealing with large population sizes, GBS-generated data are the most cost-effective choice, but the savings in genotyping costs must be balanced with increased informatics requirements.

Comparing significant QTL detected from both GWAS and interval mapping approaches were generally consistent for the detection of major genes or large-effect QTL. However, the methods differed for smaller-effect QTL, so using both approaches in combination may provide more strength of evidence for genomic regions to follow up in further validation experiments. Both new QTL and major QTL reported from independent studies were detected. Some QTL for multiple traits are within 2 cM of each other (highlighted in red in Table S3). However, in some of these cases, for example the QTL on chromosome 3 (4.19 cM; 1.27 Mbp) that is linked to both yield and flowering, the allelic effects of the founders are not alike. Further dissection of such loci, by characterizing MAGIC lines with varying allelic combinations, will help to clarify whether there are multiple QTL in this region or a single pleiotropic one. All results from our mapping effort will be moved forward for validation and marker development. The SNPseek database (Alexandrov *et al.* 2014) will be used to extract haplotypes in the region of interest.

This work aims to capitalize on the primary advantage of MAGIC populations, namely that they combine favorable alleles from several different founders for multiple traits. For geneticists, this is an ideal situation to study QTL for multiple traits using one mapping population, although increases in the number of traits and QTL rapidly add to the complexity of analysis. A systematic coordinated effort in phenotyping is critical to optimally exploit the MAGIC population. However, phenotyping and data maintenance efforts will demand time and funds. Maximizing data collected from a single trial would be an effective approach by coordinating data collection for multiple traits among phenotyping groups.

## Supplementary Material

Supplemental material is available online at http://www.g3journal.org/lookup/suppl/doi:10.1534/g3.117.042101/-/DC1.
