# Supplementary material for: Approaches in Characterizing Genetic Structure and Mapping in a Rice Multiparental Population
Source: G3 (Bethesda). 2017 Jun 5;7(6):1721–30. doi: 10.1534/g3.117.042101 (PMC5473752; doi:10.1534/g3.117.042101)
Supplement: Supplementary file 1 [file 1721FigureS1.docx]

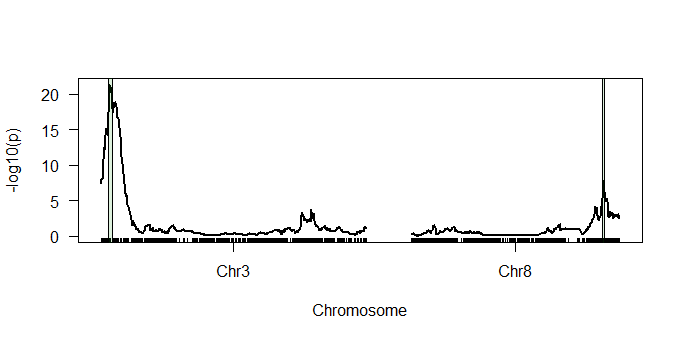


Figure S 1. Simple interval mapping output for yield (under irrigated conditions in dry season 2014 and 2015 at the IRRI experimental station) in rice - showing 1 LOD support interval for QTLs on chromosomes 3 (4.19 cM; p-value = 4.33E-22) and 8 (104.62 cM; p-value = 1.44E-08). The QTL on chromosome 3 mapped to a 72.3 kb interval (1 LOD support interval = 1.82 cM) and that on chromosome 8 mapped to a 49.2 kb interval (1 LOD support interval = 1.48 cM).
