# Supplementary material for: Approaches in Characterizing Genetic Structure and Mapping in a Rice Multiparental Population
Source: G3 (Bethesda). 2017 Jun 5;7(6):1721–30. doi: 10.1534/g3.117.042101 (PMC5473752; doi:10.1534/g3.117.042101)
Supplement: Supplementary file 2 [file 1721FigureS2.docx]

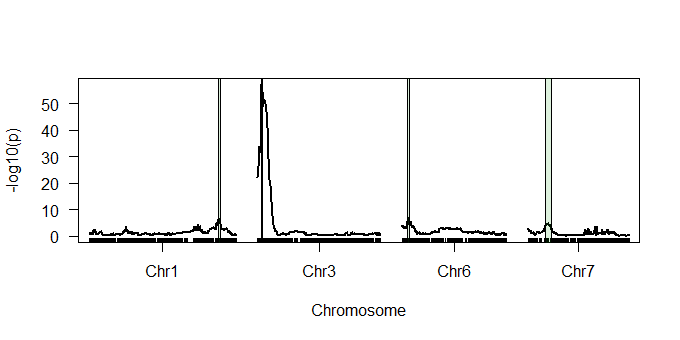


Figure S 2. Simple interval mapping output showing QTL for flowering time (2015 dry season, IRRI experimental station) on chromosomes 1 (153.1 cM; p-value = 2.59E-07), 3 (4.19 cM; p-value = 6.66E-58), 6 (7.75 cM; p-value = 1.10E-07), and 7 (23.65 cM; p-value = 1.28E-05).
