# Supplementary material for: Approaches in Characterizing Genetic Structure and Mapping in a Rice Multiparental Population
Source: G3 (Bethesda). 2017 Jun 5;7(6):1721–30. doi: 10.1534/g3.117.042101 (PMC5473752; doi:10.1534/g3.117.042101)
Supplement: Supplementary file 3 [file 1721FigureS3.docx]

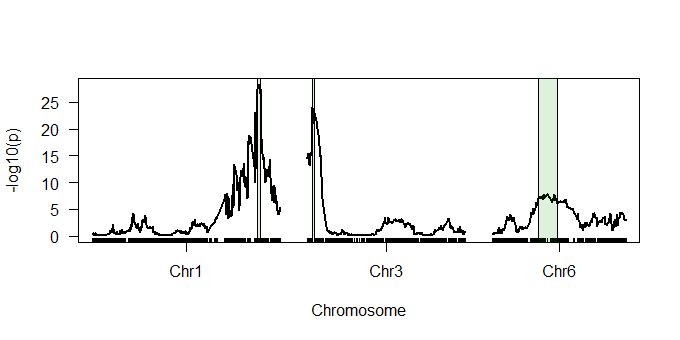


Figure S 3. Simple interval mapping output showing QTL for plant height on chromosomes 1 (153.71 cM; p-value = 5.41E-29), 3 (4.68 cM; p-value = 1.06E-24), and 6 (56.83 cM; p-value = 1.56E-08).
