# Supplementary material for: Approaches in Characterizing Genetic Structure and Mapping in a Rice Multiparental Population
Source: G3 (Bethesda). 2017 Jun 5;7(6):1721–30. doi: 10.1534/g3.117.042101 (PMC5473752; doi:10.1534/g3.117.042101)
Supplement: Supplementary file 4 [file 1721FigureS4.docx]

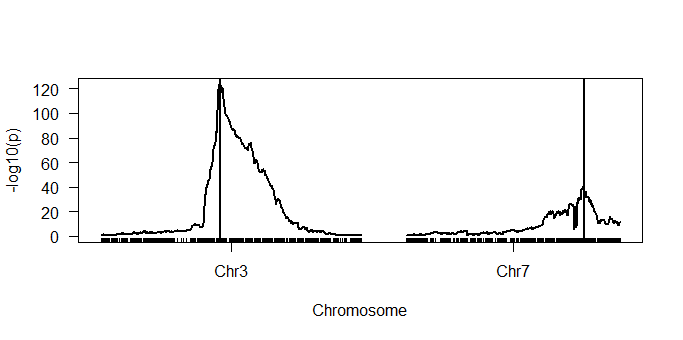


Figure S 4. Simple interval mapping output showing QTL for grain length on chromosomes 3 (65.68 cM; p-value = 3.63E-124) and 7 (98.73 cM; p-value = 1.38E-41).
