# Supplementary material for: Approaches in Characterizing Genetic Structure and Mapping in a Rice Multiparental Population
Source: G3 (Bethesda). 2017 Jun 5;7(6):1721–30. doi: 10.1534/g3.117.042101 (PMC5473752; doi:10.1534/g3.117.042101)
Supplement: Supplementary file 5 [file 1721FigureS5.docx]

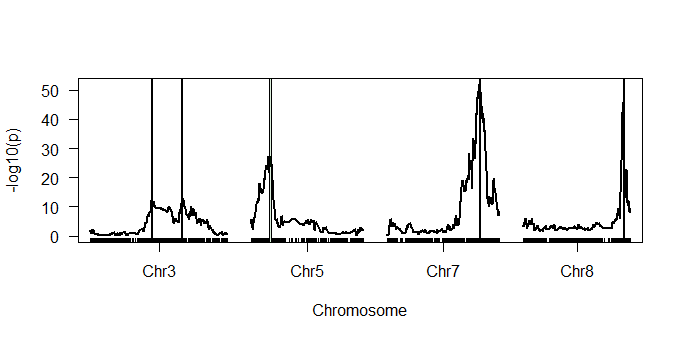


Figure S 5. Simple interval mapping output showing QTL for grain width on chromosome 3 (97.21 cM and 64.33 cM; p-value = 1.03E-14 and 6.21E-13, respectively), 5 (21.41 cM; p-value = 6.31E-28), 7 (97.6 cM; p-value = 1.36E-52), and 8 (105.95 cM; p-value = 1.64E-46) detected.
