# Supplementary material for: Approaches in Characterizing Genetic Structure and Mapping in a Rice Multiparental Population
Source: G3 (Bethesda). 2017 Jun 5;7(6):1721–30. doi: 10.1534/g3.117.042101 (PMC5473752; doi:10.1534/g3.117.042101)
Supplement: Supplementary file 6 [file 1721FigureS6.docx]

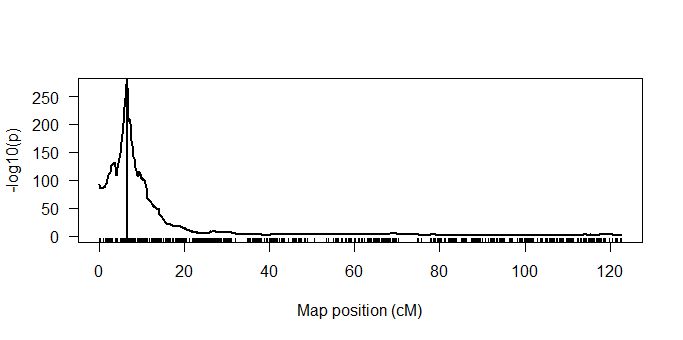


Figure S 6. Simple interval mapping output showing QTL for amylose content on chromosome 6 (6.33 cM; p-value = 5.98E-272). The waxy locus was mapped to a 1 LOD support interval of 0.27 cM and interval between flanking markers was 29.3 kb.
