# Supplementary material for: Approaches in Characterizing Genetic Structure and Mapping in a Rice Multiparental Population
Source: G3 (Bethesda). 2017 Jun 5;7(6):1721–30. doi: 10.1534/g3.117.042101 (PMC5473752; doi:10.1534/g3.117.042101)
Supplement: Supplementary file 7 [file 1721FigureS7.docx]

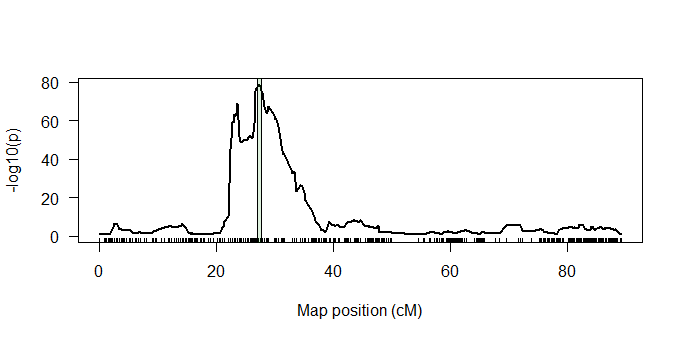


Figure S 7. Simple interval mapping output showing QTL (*SUB1A*) for submergence tolerance on chromosome 9 (27.15 cM; p-value = 1.60E-79)
