# Supplementary material for: Approaches in Characterizing Genetic Structure and Mapping in a Rice Multiparental Population
Source: G3 (Bethesda). 2017 Jun 5;7(6):1721–30. doi: 10.1534/g3.117.042101 (PMC5473752; doi:10.1534/g3.117.042101)
Supplement: Supplementary file 8 [file 1721FigureS8.docx]

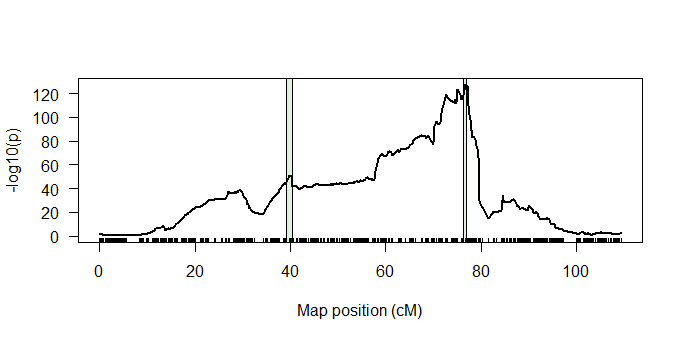


Figure S 8. Simple interval mapping output showing QTL for brown spot disease caused by Philippine isolate sm2 (seedling stage) on chromosome 12 at 39.83 cM and 76.71 cM with p-value = 2.52E-128 and 2.20E-51, respectively.
