# Supplementary material for: Approaches in Characterizing Genetic Structure and Mapping in a Rice Multiparental Population
Source: G3 (Bethesda). 2017 Jun 5;7(6):1721–30. doi: 10.1534/g3.117.042101 (PMC5473752; doi:10.1534/g3.117.042101)
Supplement: Supplementary file 9 [file 1721FileS1.docx]

**Approaches in characterizing genetic structure and mapping in a rice multi-parental population**

Chitra Raghavan, Ramil Mauleon, Vanica Lacorte, Monalisa Jubay, Hein Zaw, Justine Bonifacio, Rakesh Kumar Singh, B. Emma Huang, Hei Leung

**File S1 Guide to supplemental files and data**

Figure S1 Simple interval mapping output for yield

Figure S2 Simple interval mapping output showing QTL for flowering time

Figure S3 Simple interval mapping output showing QTL for plant height

Figure S4 Simple interval mapping output showing QTL for grain length

Figure S5 Simple interval mapping output showing QTL for grain width

Figure S6 Simple interval mapping output showing QTL for amylose content

Figure S7 Simple interval mapping output showing QTL (SUB1A) for submergence tolerance

Figure S8 Simple interval mapping output showing QTL for brown spot disease

Table S1 Summary statistics of trait across founder and in the population

Table S2 Summary table of SIM showing the allelic effect of parents

Table S3 A comparison of QTL detected by interval mapping and GWAS methods.

File S2 Details of the models used for trait analysis

File S3 Conversion between physical and genetic maps used to compute cm to bp conversion

File S4 R code to compute number of recombinations

**Guide to raw data submitted to the journal**

**Hapmap data**

https://s3-ap-southeast-1.amazonaws.com/oryzasnp-atcg-irri-org/pub-data/MAGIC-Raw-genotype-data-Raghavan-2017.zip

**VCF data**

https://s3-ap-southeast-1.amazonaws.com/oryzasnp-atcg-irri-org/pub-data/MAGIC-vcf-all-chromosomes.zip

**Genetic** **material**

Details of the lines and founders/parents used in this study are provided in the folder “Genetic material”

Number of files = 2

Material can obtained from the International Rice Research Institute, Philippines

**Raw Genotype file**

http://snpseek.irri.org/_download.zul

Raw genotype files have been provided within folder

“Raw genotype data”

Addition .xl file provides taxa names – “Taxa list”

Number of files = 13

**Raw Phenotype file**

Provided in folder

“Raw phenotype data”

Number of files = 8

**Descriptions of phenotypes**

This has been provided in the text of the manuscript

**Filtered Genotype file**

Genotype data used have been provided under the folder “Filtered genotype data”. In this folder there are 3 subfolders named below

“Data sets used for estimating recombinations”

Number of files = 18

“Genotype data used for AM”

Number of files = 1

“Genotype data used for IM” = 5

Filtering process has been detailed in the text of the manuscript (Fig1)

**Filtered and normalized Phenotype file**

Phenotype data has been provided in the following folder

“Input phenotype data for mapping”

Number of files = 8

**Results file**

Complete results for interval mapping have been provided in the manuscript

Results for AM are provided in folder “GWAS results”. Within this folder there are 8 folders and each of the folders has 2 files

**Software**
Details are provided in the manuscript
