# Supplementary material for: Approaches in Characterizing Genetic Structure and Mapping in a Rice Multiparental Population
Source: G3 (Bethesda). 2017 Jun 5;7(6):1721–30. doi: 10.1534/g3.117.042101 (PMC5473752; doi:10.1534/g3.117.042101)
Supplement: Supplementary file 10 [file 1721FileS2.docx]

**File S2:**

The models used are as below:

The linear model for augmented RCBD was used plant height, flowering time and brown spot disease.

**Equation 1: Linear Model for augmented RCBD:**

$$Y_{ij}=\mu+\tau_{i}+\rho_{j}+\epsilon_{ij}$$

Yij = observation from the jth replicate and ith lines/genotypes

µ = general mean of the response variable

τ_i_ = effect of the ith lines/genotype

ρ_j_ = effect of the jth replicate

ε_ij_ = random error associated with the jth replicate and the ith lines/genotypes

The linear model for augmented RCBD (one-stage) was used for yield analysis

**Equation 2: Linear Model for augmented RCBD (one-stage):**

$$Y_{ijk}=\mu+{\alpha_{i}+\beta}_{j}+{{\alpha\beta}_{ij}+\rho}_{k(i)}+\epsilon_{ij}$$

Yijk = observation from the jth replicate and ith lines/genotypes

µ = general mean of the response variable

α_i_ = effect of the ith environment

β_j_ = effect of the jth lines/genotype

αβ_ij_ = interaction effect of the ith environment and the jth lines/genotype

ρ_k(i)_ = effect of the kth replicate within the ith environment

ε_ij_ = random error associated with the jth replicate and the ith lines/genotypes

**Equation 3 Formula for broad sense heritability:**

H^2^ = s^2^_g_ / s^2^_p_ where s^2^_p_ = s^2^_g_ + (s^2^_e_ /r)

where s^2^_g_ – genotypic variance

s^2^_p_ – phenotypic variance

s^2^_e_ – error variance

r – no. of replicates
